# Standard Model Imaging in the Brain and Spinal Cord of MS Patients: Initial Assessment and Comparison to Diffusion Tensor Imaging

**DOI:** 10.1002/nbm.70354

**Published:** 2026-07-08

**Authors:** Atlee Witt, Alicia E. Cronin, Bailey Busher, Isabella Stuart, Grace Sweeney, Kristin P. O'Grady, Seth A. Smith, Samantha By, Kurt Schilling

**Affiliations:** ^1^ Vanderbilt University Institute of Imaging Science Nashville Tennessee USA; ^2^ Vanderbilt University School of Medicine Nashville Tennessee USA; ^3^ Department of Radiology and Radiological Sciences Vanderbilt University Medical Center Nashville Tennessee USA; ^4^ Department of Biomedical Engineering Vanderbilt University Nashville Tennessee USA; ^5^ Bristol Myers Squibb Lawrenceville New Jersey USA

**Keywords:** brain, diffusion models, diffusion‐weighted imaging, multiple sclerosis, spinal cord

## Abstract

Multiple sclerosis (MS) pathology manifests across the central nervous system (CNS), yet magnetic resonance imaging (MRI) studies frequently assess the brain and spinal cord (SC) in isolation. It remains unclear whether microstructural injury in normal‐appearing white matter (NAWM) and lesions manifests identically across these distinct anatomical environments or if advanced biophysical models offer diagnostic advantages over standard diffusion techniques. We performed a comprehensive 3T MRI protocol with same‐session brain and cervical SC imaging on 34 persons with relapsing–remitting MS (pwRRMS) and 36 healthy controls (HCs). We focused on diffusion frameworks (diffusion tensor imaging [DTI], standard model imaging with free water [SMIfw]) to quantify and compare microstructural integrity between the brain and cervical SC for the first time. Specifically, we assessed the feasibility of SMI for clinical implementation in terms of sensitivity (contrast between lesions and NAWM in pwRRMS) and reliability (HC reproducibility). Our findings indicate that both simple (DTI) and advanced (SMI) models are sensitive to MS‐related microstructural changes, but the utility varied by region: notably, SMI‐derived neurite density fraction (*f*) emerged as a robust marker of MS pathology in both the brain and SC, while DTI metrics like fractional anisotropy (FA) and radial diffusivity (RD) showed comparable sensitivity exclusively in the brain. Though axonal loss appears to be a global feature detectable even in early‐stage disease, the inflammatory or edematous environment of lesions differs fundamentally between the brain and SC. Taken together, these findings demonstrate the utility of SMI for comprehensive CNS assessment and support its potential for future usage in clinical trials and improved disease monitoring.

## Graphical abstract

DTI and SMIfw diffusion models applied in persons with multiple sclerosis revealed region‐specific sensitivity to microstructural injury, but performance differed by anatomical region. SMIfw neurite density fraction demonstrated high sensitivity and reliability in both the brain and spinal cord, whereas DTI measures such as fractional anisotropy and radial diffusivity showed comparable reliability only in the brain. These findings indicate global axonal injury and highlight SMIfw as a feasible marker for CNS‐wide assessment.

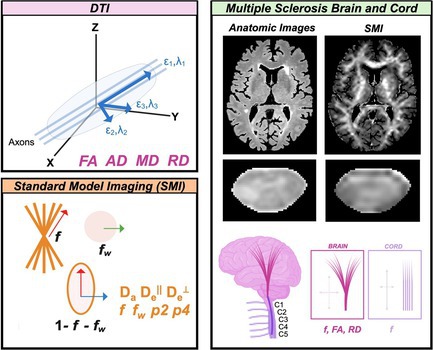

## 1 Introduction

Multiple sclerosis (MS) is a complex neurological disease characterized by inflammation, demyelination, and axonal loss throughout the central nervous system (CNS). While conventional magnetic resonance imaging (MRI) is the diagnostic clinical standard, its utility is limited. T1‐ and T2‐weighted scans can visualize focal lesions but have low sensitivity to microscopic damage occurring in normal‐appearing white matter (NAWM) [1, 2, 3]. This hidden pathology is thought to be a key driver of irreversible disability, revealing a critical need for advanced imaging techniques that can noninvasively quantify tissue injury in MS. Furthermore, adequate assessment of disease across the CNS is essential, as MS pathology in the brain and spinal cord (SC) may not present concurrently and is often poorly correlated [4, 5, 6] with implications for anatomic‐specific treatment and monitoring strategies.

Diffusion MRI offers a window into the CNS microstructural environment by measuring the random motion of water molecules [7]. While models like diffusion tensor imaging (DTI) are sensitive to pathological changes, biophysical models like standard model imaging (SMI) provide greater biological specificity by separating the diffusion signal into distinct tissue compartments [2, 8, 9]. Many biophysical models share an overarching framework [1, 10, 11, 12] but apply subtly different biophysical feature assumptions to characterize tissue microstructure; for example, SMI directly estimates features such as neurite density fraction (*f*), a marker of axonal and dendritic density, and free‐water fraction (*f*_*w*_), which reflects edema or neuroinflammation [8, 13]. This requires additional parameters “p2” to characterize the orientation and degree of axon bundles dispersion and “p4” to capture higher‐order deviations from isotropic diffusion. These indices have been shown to be highly specific to normal anatomical variation [14] and disease processes such as demyelination [15, 16], axonal loss (*f*) [16], and axonal beading [17].

SMI has been successfully applied to study the brain in persons with MS (pwMS), revealing microstructural abnormalities like reduced neurite density and higher perpendicular extracellular diffusion (D_e_^⊥^) compared with healthy controls (HCs) [2]. These findings track with MS disease severity according to a patient determined questionnaire, suggesting SMI microstructural measures may reflect ongoing histopathologic changes [2]. However, there has yet to be an investigation of SMI in the MS SC, and very few studies have performed comprehensive diffusion imaging of both the brain and SC during a single session [18, 19, 20]. It remains unclear whether the SMI microstructural signatures of MS are uniform throughout the CNS or if the disease manifests with divergent pathological features in the brain compared with the highly organized SC. A feasible next step is to assess the reproducibility and sensitivity of SMI in the SC to determine whether it is comparable to that of the brain.

To address this gap, we implemented a comprehensive, diffusion MRI protocol using DTI and SMI‐free water (SMIfw) for same‐session imaging of the brain and cervical SC in persons with relapsing–remitting MS (pwRRMS) and HCs. Our primary goals were (1) to identify diffusion measures sensitive for detecting differences between MS lesions and NAWM and between NAWM and healthy white matter (WM) and (2) to quantify the reliability of diffusion‐derived metrics for conventional DTI and advanced SMI diffusion models. By doing so, we aim to comprehensively assess the feasibility of advanced diffusion in characterizing microstructural signatures across the CNS, with the goal of identifying robust imaging biomarkers for clinical trials.

## 2 Methods

### 2.1 Data Acquisition

Collection of anatomical and diffusion data was approved by the Vanderbilt Institutional Review Board Health Sciences Committee and performed in accordance with relevant ethical guidelines. Inclusion criteria for persons with MS (pwMS) included relapsing–remitting disease and no contraindications to 3T MRI [21]. Studies were performed in 36 HCs and 34 pwRRMS (Table 1) with only complete cases (both brain and cord data acquired) included for the purpose of this analysis.

**TABLE 1:** Demographic averages with standard deviation for HCs and pwRRMS. Age and disease duration are reported as the average ± the standard deviation. Expanded Disability Status Scale (EDSS) is reported as the median (range).

	HCs (*n* = 36)	pwRRMS (*n* = 34)	*p*
Sex at birth	26F/10M	29F/5M	NS
Age (years)	38.49 ± 12.51	42.09 ± 9.50	NS
EDSS (median score)	—	2(0–6)	
Disease duration (years)	—	12.05 ± 8.86	

Participants were scanned on a 3T Philips Elition X (Philips Medical Systems, Best, The Netherlands) using a dual‐channel transmit body coil and 16‐channel neurovascular coil for signal reception centered in the cervical SC at C3 to C4 to encompass C2–C5 cervical vertebral levels. No gadolinium‐based contrast agent was administered. Repeat scans were performed at least 1 week following the initial scan using an identical protocol.

Brain acquisition (Table 2) was aligned in plane with the anterior commissure–posterior commissure line, with rotation around the L‐R axis to ensure full field of view. Cord acquisition is included in Table 3. Both brain and cervical cord images were acquired during a single MRI session. The protocols were optimized for either brain or SC anatomy rather than harmonizing the acquisition across anatomies, as this provided tailored, high‐quality imaging per individual region.

**TABLE 2:** Scan parameters for the brain. Diffusion data were collected using single‐shot echo‐planar imaging (EPI) readout, with reduced SC field of view (FOV) using the outer volume suppression technique and spectral pre‐saturation inversion recovery (SPIR) method for fat suppression. Diffusion sensitization included *b*‐values 750/1500/2250/3000 s/mm^2^ acquired with 10/20/30/40 diffusion directions per shell, and 10 measurements at *b*‐value 0 s/mm^2^. Diffusion timing parameters for both brain and cord acquisitions were 21 ms (*δ*) and 41 ms (∆). The sagittal T2 fluid‐attenuated inversion recovery (FLAIR) sequence included a reconstructed voxel size of 0.89 × 0.89 × 1 mm^3^. AP, anterior–posterior; T1w, T1‐weighted anatomical scan; TR/TE, repetition time/echo time.

Sequence	TR/TE (ms)	α	Resolution (mm^2^)	Slice (mm)	FOV (mm^2^)	SENSE (AP)	Slices	Time (min)
Single‐shot EPI diffusion	4600/85	—	2.5	2.5	96 × 94	2.2	62	9:16
T2 FLAIR	4800/1650	40°	1	1	256 × 256	—	220	4:33
T1w	6.3/2.9	8°	1	1	256 × 240	—	225	5:37

**TABLE 3:** Scan parameters for the SC, using a similar diffusion sequence as the brain. The sagittal T2‐weighted turbo spin echo (TSE) sequence included a reconstructed voxel size of 0.5 × 0.5 × 2 mm^3^. The multi‐echo fast field echo (mFFE) included a reconstructed voxel size of 0.31 × 0.31 × 5 mm^3^.

Sequence	TR/TE (ms)	α	Resolution (mm^2^)	Slice (mm)	FOV (mm^2^)	SENSE (AP)	Slices	Time (min)
Single‐shot EPI diffusion	4600/85	—	1.11	5	72 × 51	1.8	18	9:16
T2w TSE	2500/100	90°	0.8 × 1	2	160 × 250	—	18	2:30
mFFE	700/8	28°	0.65	5	160 × 160	—	14	5:47

### 2.2 Anatomical Image Processing

The image processing steps were adapted from prior work [9, 22] utilizing the Spinal Cord Toolbox (SCT) [23] and FSL [24, 25]. Brain segmentations were obtained via FreeSurfer 6.0 [26] to derive WM and gray matter (GM) regions‐of‐interest (ROIs). Cord segmentations were obtained following vertebral labelling on the mFFE and registration to the PAM50 template to produce WM and GM ROIs. Automated lesion segmentation via SAMSEG [27] and *sct_deepseg_sc* was applied to the T2 FLAIR/non‐contrast T1w (brain) and mFFE (SC), respectively, to highlight hyperintensities. All lesion voxels across a given subject were pooled into a single representative value.

Brain volume was calculated by summing the binary voxels within the WM and GM masks. Cord area was calculated via *sct_process_segmentation* to capture the cross‐sectional area (CSA) across spinal levels C3 and C4, henceforth referred to as brain and SC morphometry.

### 2.3 Diffusion Imaging Processing

Brain diffusion preprocessing was performed using the FSL and MRTrix3 software, including Marcenko‐Pastur PCA denoising [28], Gibbs‐Ringing correction [29], and correction of motion, eddy currents, and susceptibility distortion [30, 31] followed by denoising using Patch2Self [32].

Cord diffusion preprocessing was performed using Marcenko‐Pastur PCA denoising, Gibbs‐ringing correction, and motion correction using *sct_dmri_moco* [33] with parameters: *‐g 10 ‐param metric = CC smooth = 0* to address high *b*‐value data and improve signal‐to‐noise ratio (SNR). Following motion correction and Patch2Self denoising [34], the motion‐corrected images were quality checked, with grouping, metric, smoothing, and step parameters changed as needed based on magnitude and frequency. More specifically, these manual adjustments included changing the grouping parameter (*−g* in *sct_dmri_moco*) during motion correction and/or changing the iterations and gradient step (*−iter* and *‐gradStep* in *sct_register_multimodal*) to enable more or less parameter space in registration.

### 2.4 Diffusion Measure Modeling and Definitions

**FIGURE 1:**
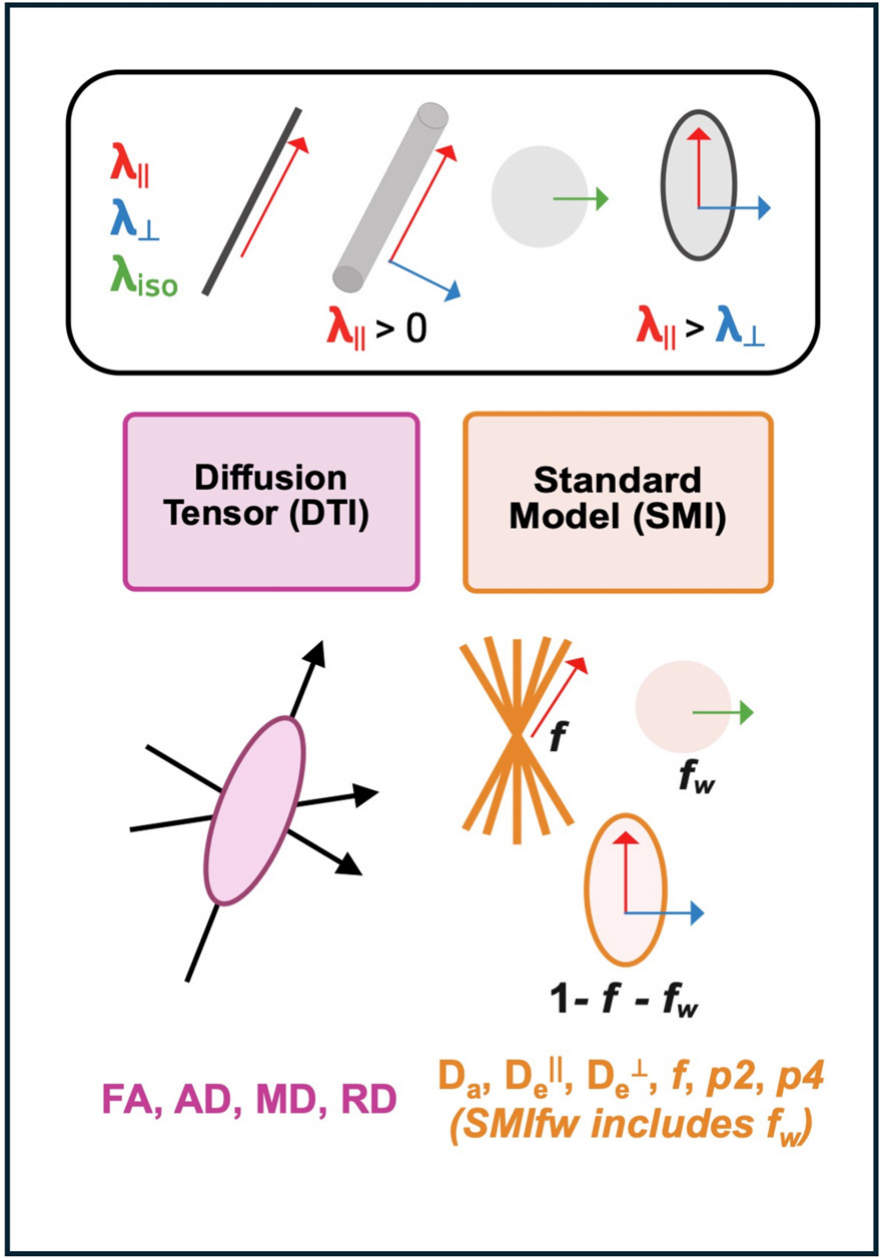
All diffusion models can be roughly summarized similar concepts, which can be captured either by “sticks” or cylinders, spheres with isometric diffusion, or diffusion tensors with directional diffusion. In this depiction, SMI represents both SMI and SMIfw models, with SMIfw including a *f*_*w*_ measure not otherwise included in the SMI model.

DTI was fit using the FSL toolbox *dtifit*, fitting the diffusion tensors using linear least squares on the log‐transformed signal and resulting in fractional anisotropy (FA), axial diffusivity (AD), radial diffusivity (RD), and mean diffusivity (MD) maps. SMI fitting was performed using the SMI MATLAB toolbox from NYU [8], with specific emphasis on the SMIfw model (Figure 1). The acronyms used throughout this analysis and in the figures are included in Table 4.

**TABLE 4:** Measures included in the DTI and SMIfw models and their associated acronyms applied in this analysis. AD, axial diffusivity; *D*_*a*_, intra‐axonal diffusivity; *D*_*e*_^||^, extracellular parallel diffusivity; D_e_^⊥^, extracellular perpendicular diffusivity; *f*, neurite density fraction; FA, fractional anisotropy; *f*_*w*_, free‐water fraction; MD, mean diffusivity; p2, characterizes orientation and degree of axon bundle dispersion; p4, captures higher‐order deviations from isotropic diffusion; RD, radial diffusivity.

Model	Measure	Acronym
DTI	FA, AD, RD, MD	FA, AD, RD, MD
SMIfw	D_a_, D_e_^||^, D_e_^⊥^, *f*, p2, p4, *f*_*w*_	Da, DePar, DePerp, *f*, p2, p4, *f*_*w*_

### 2.5 Statistical Analysis

R (v4.4.0; R Core Team, 2024) was used for all analyses. Demographic and clinical variables were compared with Welch's *t*‐tests (continuous) and chi‐squared tests (sex at birth). Diffusion measures were analyzed via linear models or *t*‐tests.

For between‐group comparisons (HC WM versus MS NAWM; HC *n* = 36, MS *n* = 34), we fit an unpaired linear model: *Normalized Measure ~ Group + Age + Sex + Morphometry*, where morphometry represented the regional volume. For within‐group comparisons (MS NAWM versus MS lesions; MS *n* = 34), we used a linear mixed‐effects model: *Normalized Measure ~ Group + Age + Sex + Morphometry + (1|Subject)*, with a random intercept to account for repeated subject measures. Measures and morphometry were *z*‐scored per measure across all subjects prior to modeling, and the standardized regression *β* coefficient reflected the estimated group difference. FDR‐adjusted *p*‐values were reported to account for multiple comparisons across measures, with significance determined as *p* < 0.050. For *t*‐tests, significance was determined as *p* < 0.050 with Bonferroni correction for between‐group comparison independent *t*‐tests (HC WM versus MS NAWM; HC WM versus MS lesion) and within‐group comparison paired *t*‐tests (MS NAWM versus MS lesion). Three pwRRMS with SC lesion volumes of 0 were excluded from the paired *t*‐test analysis between NAWM and lesions.

For the sensitivity‐reliability analysis, the standardized regression coefficient for NAWM versus MS lesion contrast was associated with the scan‐rescan reliability of HC WM (scan‐rescan HC *n*  = 21), quantified using a two‐way random effect, single measure, absolute agreement intraclass correlation coefficient (ICC). Scan‐rescan reliability was assessed exclusively in the HC cohort to preclude confounding by disease‐driven biological factors in the MS population. The “composite score” was calculated as the absolute effect size of the *β* coefficient, adjusted for age, sex, and regional volume, multiplied by the ICC score with equal weighting. This score was intended to identify measures that may be useful for clinical application, where both sensitivity to pathological tissue differences and scan–rescan reliability are important.

For the brain, ICC below 0.5 was considered poor reliability, 0.5 to 0.75 as moderate reliability, and above 0.75 as good reliability [9, 35, 36]. For the SC, more relaxed criteria were implemented, given ICC may be lower in the SC compared with the brain [9] as a result of motion, susceptibility, and lower SNR [37]. Further, attempts to improve repeatability in the SC must be balanced with longer acquisition times that may inherently contribute to additional subject motion. Thus, ICC above 0.5 was considered acceptable reliability in the SC based on existing literature.

## 3 Results

### 3.1 Participant Demographics

Demographic variables were not significantly different between HCs and pwRRMS.

### 3.2 Brain and Cord Morphometry

**FIGURE 2:**
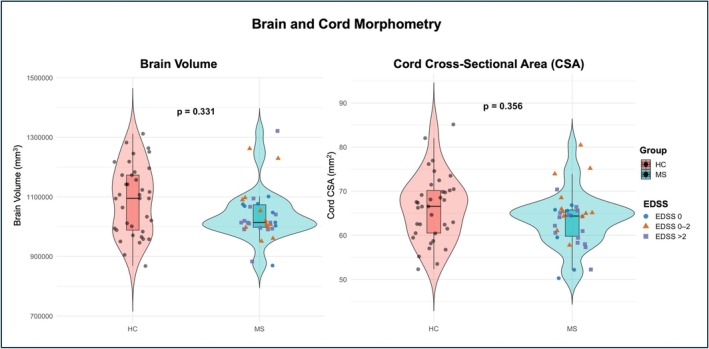
Brain and cord morphometry compared between HCs and pwRRMS following correction for age and sex. No significant relationships were noted (*p* < 0.050), though MS morphometry trended lower than HC morphometry.

There were no noted significant differences for brain and cord morphometry, respectively, between HCs and pwRRMS after correcting for age and sex (Figure 2). Younger participants (*β* = −2657, *p* = 0.005) and males (*β* = 146,985, *p* < 0.001) had significantly greater brain volumes compared with their counterparts, and males (*β* = 6.278, *p = 0.002)* had significantly greater cord area.

### 3.3 Brain and SC SMIfw Maps

**FIGURE 3:**
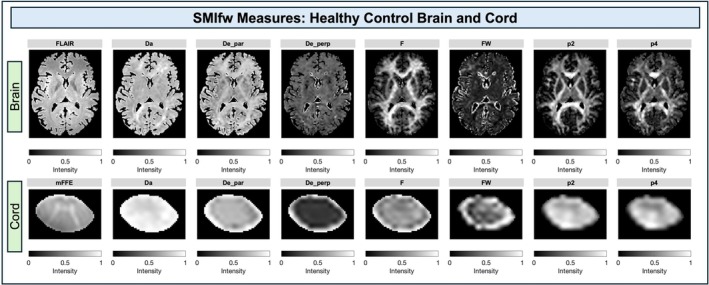
All measures captured by in the SMIfw diffusion model for one HC (28.9‐year‐old female) in the brain and SC. The T2 FLAIR and mFFE images were normalized between 0 and 1 for visualization and included as anatomical references. For diffusion metrics, intensity values within brain and cord regions were independently normalized by their respective 99th percentile values to scale diffusion maps between 0 and 1 for visualization.

**FIGURE 4:**
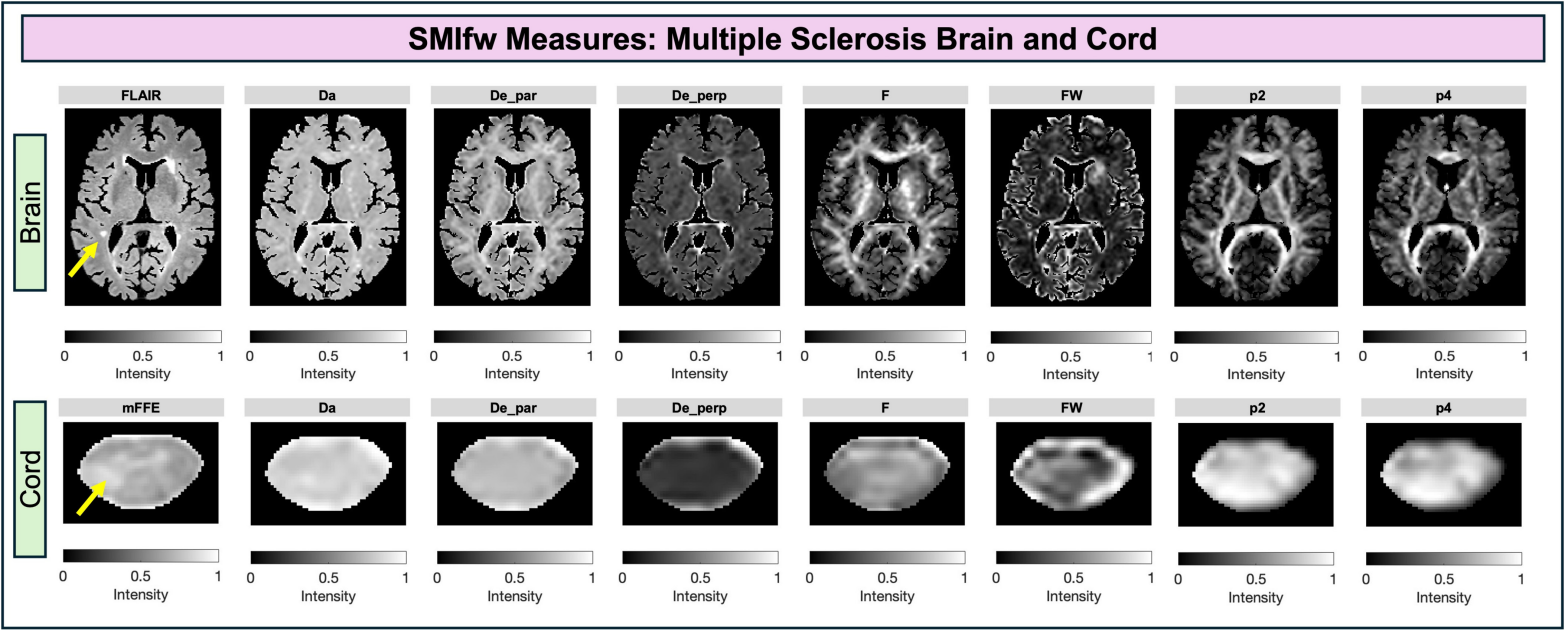
All measures captured by in the SMIfw diffusion model for one pwRRMS (58.5‐year‐old male) in the brain and SC. The FLAIR and mFFE images were normalized between 0 and 1 for visualization and included as anatomical references, with lesions identified via the yellow arrow. For diffusion metrics, intensity values within brain and cord regions were independently normalized by their respective 99th percentile values to scale diffusion maps between 0 and 1 for visualization.

Examples of the SMIfw model features in an HC and pwRRMS are included in Figures 3 and 4, respectively. In the HC example, the GM/WM boundary was most pronounced on *f* (brain) and p2/p4 (cord) maps. Residual signal related to cerebrospinal fluid (CSF) was visualized outside the cord, particularly on the *f*_*w*_ map likely from partial volume effects.

In the pwRRMS example, the right temporal brain lesion was visible across multiple contrasts, with notably reduced *f,* p2, and p4, and increased *f*_*w*_ and D_e_^⊥^ within the lesion compared with the surrounding normal‐appearing tissue. While the SC right lateral lesion was less pronounced on D_e_^⊥^ or D_e_^||^, the lesion was apparent on other maps like *f* and *f*_*w*_, with similar findings as the brain of reduced *f* and increased *f*_*w*_ and D_e_^⊥^ compared with normal‐appearing tissue.

### 3.4 Sensitivity‐Reliability Analysis

#### 3.4.1 Healthy WM Versus MS NAWM

In both the brain and SC, there were evident trends of reduced anisotropy, reduced *f*, and increased *f*_*w*_ in MS NAWM compared with HC WM. When comparing the *β* effect sizes for normalized measures between HC WM and MS NAWM, there were no significant differences in the brain following FDR correction (Figure S1). The greatest effect size in the brain was *f* (HC WM > MS NAWM; absolute *β* = 0.523). In the SC, there was significantly decreased FA (absolute *β* = 0.667, *p* = 0.047) in MS NAWM compared with HC WM following FDR correction (Figure S1).

#### 3.4.2 MS NAWM Versus MS Lesions: Brain

There were significant differences in multiple measures between pwRRMS NAWM and MS lesions, with all measures in the brain demonstrating significance following FDR correction (Figure S2). There was significantly decreased *f* (absolute *β* = 1.666, *p* < 0.001), increased *f*_*w*_ (absolute *β* = 1.800, *p* < 0. 001), decreased FA (absolute *β* = 1.750, *p* < 0.001), and increased diffusivities captured by both the DTI and SMIfw models in MS brain lesions compared with MS NAWM. The greatest effect size difference was D_e_^⊥^ (MS lesion > MS NAWM; absolute *β* = 1.878, *p* < 0.001).

**FIGURE 5:**
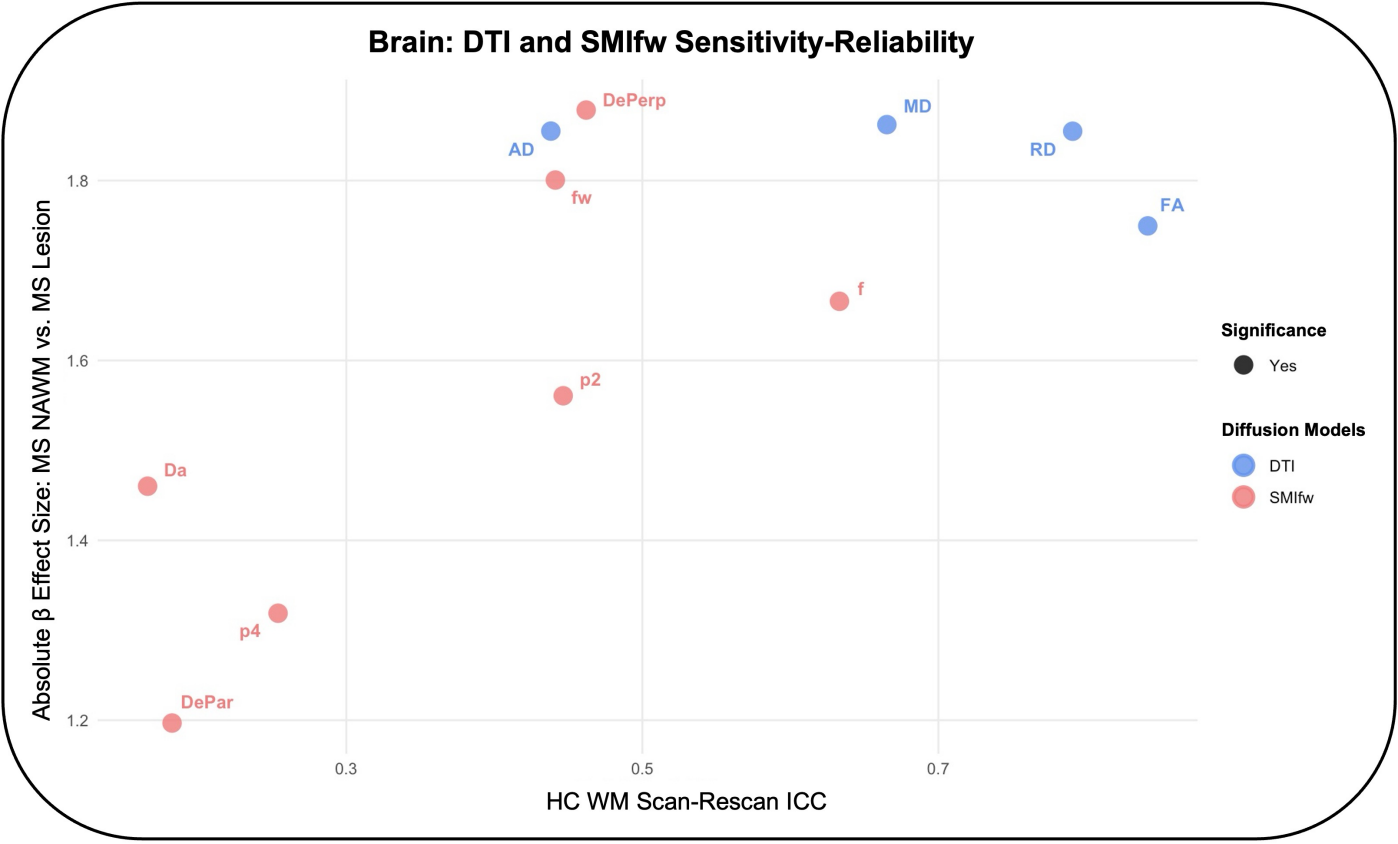
Sensitivity–reliability analysis in the brain using absolute β effect size between MS NAWM versus MS lesions and HC WM scan‐rescan ICC. The blue markers represent measures from the DTI model, while the red markers represent measures from the SMIfw model. Significance (filled‐in marker) is based on the FDR‐corrected *p*‐values from the absolute *β* effect size measurements.

For clinical utility, a balance between meaningful contrast and robustness must be achieved. When comparing sensitivity (absolute *β* effect size) with reliability (HC ICC) in the brain for measures captured by the DTI and SMIfw models, measures with high composite scores included RD (absolute *β* = 1.855, ICC = 0.790), FA (absolute *β* = 1.750, ICC = 0.841), MD (absolute *β* = 1.862, ICC = 0.665), and *f* (absolute *β* = 1.666, ICC = 0.633) (Figure 5).

#### 3.4.3 MS NAWM Versus MS Lesions: Cord

**FIGURE 6:**
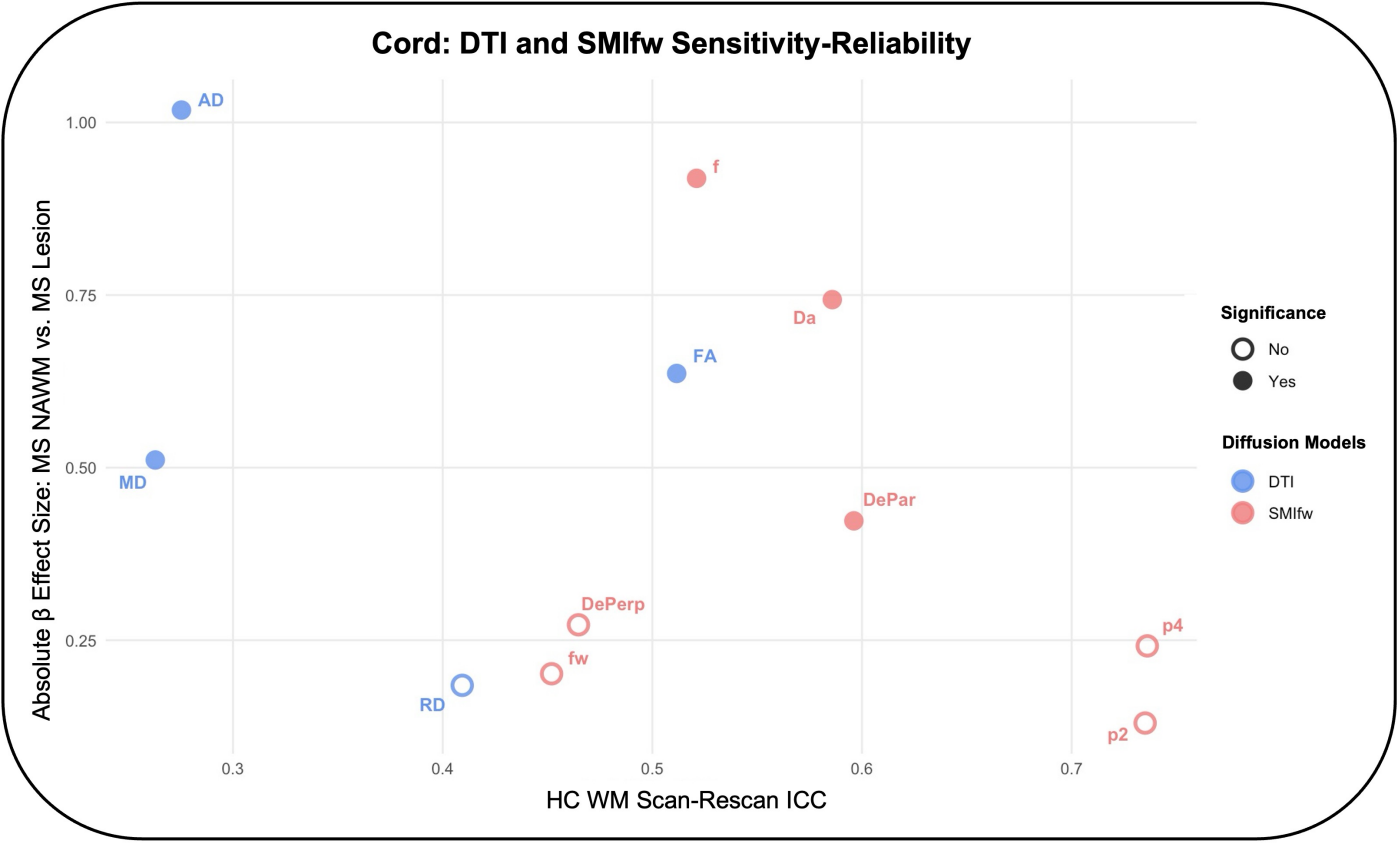
Sensitivity–reliability analysis in the SC using absolute *β* effect size between MS NAWM versus MS lesions and HC WM scan‐rescan ICC. The blue markers represent measures from the DTI model, while the red markers represent measures from the SMIfw model. Significance is based on the FDR‐corrected *p*‐values from the absolute *β* effect size measurements.

There was significantly decreased *f* (absolute *β* = 0.920, *p* < 0.001), decreased FA (absolute *β* = 0.636, *p* = 0.010), and decreased diffusivities like AD (absolute *β* = 1.018, *p* < 0.001) in SC MS lesions compared with MS NAWM (Figure S2). The decrease in AD represented the greatest effect size difference between MS NAWM and lesions. For the effect size and ICC analysis in the cord, measures with high composite scores included *f* (absolute *β* = 0.919, ICC = 0.521) and D_a_ (absolute *β* = 0.743, ICC = 0.585) (Figure 6).

### 3.5 HC WM, MS NAWM, and MS Lesion Diffusion Value Comparison

**FIGURE 7:**
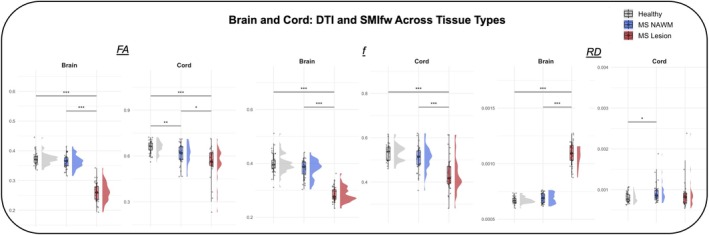
Comparison of FA, *f*, and RD diffusion values in the brain and SC with Bonferroni *p*‐value correction. *< 0.050, **< 0.010, ***< 0.001.

From the sensitivity‐reliability findings, FA, RD, and *f* were selected for raw‐value comparison as representative measures of anisotropy, diffusivity, and neurite density. For FA in the brain, both HC WM and MS NAWM were significantly greater than MS lesion values (*p* < 0.001). In the cord, FA in HC WM was significantly greater than MS NAWM (*p* = 0.003) and MS lesions (*p* < 0.001), and FA in MS NAWM was significantly greater than MS lesions (*p* = 0.015). Similarly, *f* in both HC WM and MS NAWM was significantly greater than MS lesion values in both the brain and the cord (*p* < 0.001). For RD, both HC WM and MS NAWM in the brain were significantly diminished compared with MS lesions (*p* < 0.001), while only HC WM in the cord was significantly diminished compared with MS NAWM (*p* = 0.037) (Figure 7).

## 4 Discussion

We sought to explore simple (i.e., DTI) and biophysical (i.e., SMIfw) diffusion models, quantify both the sensitivity and reliability of associated measures, and evaluate the microstructural environment of different tissues in both pwMS and HCs in the brain and SC. For the first time, we successfully applied SMIfw modeling to the MS SC to capture specific pathologic features like *f* and *f*_*w*_. By probing the heterogeneous landscape of MS and leveraging measures derived from diffusion models such as SMIfw, this may present an opportunity to bridge the microstructure‐disability gap in MS when correlated with clinical disability in future studies.

### 4.1 Brain and Cord Morphometry Does Not Differ Between HCs and pwRRMS

We did not find a difference in either brain or cord morphometry between HCs and pwRRMS. Longitudinal studies in the brain and upper cervical SC establish MS tissue loss, particularly in the early phase of the disease with disease durations less than 6 (brain) and 5 (cord) years [38]. Our lack of significance may be related to our low‐disability cohort, and that we did not capture longitudinal changes over time. The significantly greater brain volume in males and younger participants is established [38], and there are regional differences in brain volume between males and females that were not captured by our total WM and GM masks [39, 40]. There is also evidence of variation in SC sizes due to age and sex [41] and increased SC CSA and anteroposterior and transverse SC diameters in males compared with females [42, 43] supported by our work.

### 4.2 Models Converge on Reduced *f*, Increased *f*_*w*_, and Reduced Anisotropy in MS NAWM Versus HC WM

The application of advanced diffusion imaging in the SC is complicated by numerous factors, including its substantially smaller average diameter of 1 cm compared with the brain. Marked differences in magnetic susceptibility between bone, air, and fluid‐filled structures like vertebral discs contribute to local field inhomogeneities, and partial volume effects from the small SC size are often significant [44]. Additionally, the dynamic nature of the SC with substantial physiologic noise and bulk motion must be accounted for [45]. These factors make localization of SC signal changes at a voxel or sub‐voxel level challenging, though multi‐compartment models like SMI provide an opportunity to describe the microstructural environment in greater detail [9]. Our study contributes to a growing number of investigations applying multi‐compartment diffusion models in the SC, with notable novelty using comprehensive brain and SC imaging, and the first application of SMIfw in the SC. We chose to focus our regional analysis in NAWM to highlight the potential advantages of advanced diffusion over conventional imaging in identifying areas of subtle microstructural changes preceding lesion formation.

In both the brain and SC, reduced *f* in MS NAWM was a nonsignificant though robust finding, suggesting WM microstructural abnormalities in the MS CNS precede and exist outside of just macrostructural damage. While reduced *f* has been established in brain NAWM [2], the finding has yet to be identified in SC NAWM using the SMIfw model. We provide corroborating evidence of reduced *f*, suggestive of axonal and dendritic loss, in two distinct anatomies [46].

We also identified a similar trend of a nonsignificant *f*_*w*_ increase in MS NAWM compared with HC WM in the brain and SC. While myelin‐water imaging is commonly used to assess CNS pathology in pwRRMS [47], our study is one of the first to assess *f*_*w*_ derived from SMIfw across the CNS. The observation of increased *f*_*w*_ in the brain of pwRRMS aligns with previous work [48], with pathologic processes in the MS CNS linked to enlarged perivascular spaces [49]. Taken alongside reduced *f* in NAWM, we interpret our results as evidence of reduced axonal integrity and diminished cellularity across the MS CNS, and notably, in tissues that have yet to demonstrate radiological evidence of lesions.

It is worth noting that the findings from conventional diffusion were as expected. A significantly diminished SC FA in MS NAWM compared with HC WM was observed in both the between‐group analysis and the raw‐value analysis [22, 50], as well as the associated lack of FA significance when comparing brain NAWM in pwRRMS to healthy tissue [51].

### 4.3 MS Lesions Demonstrate Reduced Neurite Density Compared With MS NAWM, With Divergent Signatures in the Brain and the SC

For the intra‐subject analysis comparing measures in MS lesions with MS NAWM, evidence of significant differences between MS lesions and MS NAWM was stark. In the brain and SC, a significantly reduced *f* in MS lesions compared with MS was observed; however, only a significant increase in the *f*_*w*_ measure in MS brain lesions was observed, in addition to a broad increase in diffusivity in brain lesions not seen in the SC. Taken together, these findings point to divergent pathological signatures depending on anatomical region.

Our findings infer a more complex anatomical organization of the SC, benefitting advanced diffusion models to parse out microstructural changes. This is corroborated by our previous work, where we have observed brain lesions frequently visible on conventional T1‐ or T2‐weighted sequences, whereas SC lesions often go undetected with these water‐sensitive modalities irrespective of resolution. In contrast, our use of mFFE T2*‐weighted imaging is more effective at capturing subtle pathological changes in the SC, providing evidence for the need for tailored imaging strategies for the brain and SC. Such a shift may help resolve aspects of the clinico‐radiologic paradox by revealing microstructural and macrostructural damage that conventional imaging fails to detect [52].

Diminished tissue coherence is expected to be reflected by decreased anisotropy (i.e., FA, p2 and p4), as observed in the brain/SC between‐group analysis and SC within‐group analysis. In contrast, we observed reduced FA and increased p2/p4 in MS lesions compared with MS NAWM. This may be due to subtle changes in NAWM that have not been clearly resolved on conventional imaging. Furthermore, decreases in p2 in pwMS may be associated with the neuroinflammatory response and microglial activation [2, 52]. While this is supported by increased *f*_*w*_ measures in MS brain lesions, we did not differentiate lesions based on disease activity.

### 4.4 Simple Diffusion Models May Provide Useful Information on Par With More Advanced Models, Though the Utility May Differ Depending on Anatomic Region

The categorization of “simple” or “biophysical” diffusion models leaves room for interpretation, as there is no one defining cut‐off between these descriptors. Even so, it is possible to distribute diffusion models along this rough scale. In the brain, “simpler” models like MD, RD, and FA had the highest composite scores when combining absolute effect size with scan‐rescan reliability, representing robust combined sensitivity to damage and reliability across two timepoints.

However, in the SC, *f* exhibited the highest sensitivity‐reliability composite score, with DTI metrics demonstrating comparatively lower ICC values than those observed in the brain. Postmortem assessments of the human MS SC reveal extensive axonal loss [4, 53] that may not be fully captured by changes in conventional diffusion measures like AD [54]. Our observations suggest SMI‐derived *f* may serve as a biomarker of axonal loss in the SC, where reductions in axon density may lead to proportionally larger shifts in *f* given the lower baseline axon count relative to the brain.

It remains important to consider whether neurite compartments in the SC satisfy the SMI assumption of zero‐radius cylinders [9] before such interpretations can be fully validated. While simpler diffusion models such as DTI may be sufficient to sensitively and reliably detect microstructural alterations in the brain, the distinct anatomy of the SC and the inherent challenges of imaging the SC may require compartment‐specific diffusion approaches. As SC‐specific validation is currently limited, our intention with this exploratory study was to utilize tools that are readily available for SMIfw processing and apply them to the MS SC for the first time. Formal validation of SC‐specific model assumptions is a natural next step. We believe our neurite‐focused SC observations suggest SMIfw measures may be consistent with changes in pathology, as direct validation of axonal density is outside the scope of this investigation.

This study highlights differences between the brain and SC microenvironments that are not fully captured by a single diffusion model; further work is needed to characterize these differences in greater detail. Biophysical diffusion frameworks may provide nuanced information about measures related to pathologic processes like axonal loss, though models like DTI may still be sensitive to tissue changes if limited by post‐processing or imaging capabilities. This must be considered when implementing a diffusion protocol for a multi‐site clinical trial, where scanner manufacturers, hardware, and site expertise may vary.

### 4.5 Limitations

We acknowledge several limitations in this study. First, the RRMS cohort was limited to participants with minimal disability (average EDSS = 2) and on disease modifying treatments. It would be worthwhile to expand this to a cohort with increased disability. Future work should focus on assessing longitudinal changes in diffusion measures within both MS NAWM and lesions and incorporating measures of sensorimotor dysfunction to evaluate associations with clinical outcomes.

Additionally, we recognize that conventional DTI is driven primarily by low‐to‐moderate *b*‐value signal and that a comparison between model‐based SMIfw and representation‐based diffusion kurtosis imaging (DKI) may offer a more informative benchmark. In a post hoc analysis incorporating DTI, DKI, and SMIfw (Figures S3 and S4), the addition of DKI further supports our conclusions regarding anatomically sensitive diffusion measures and does not alter our primary findings.

Other limitations include long diffusion scan times (> 9 min per brain/SC) that risk increased motion, decreased SNR, and increased sensitivity to exchange, though previous work with similar acquisition parameters and post‐processing techniques demonstrated success in capturing high‐fidelity images with sufficient WM/GM contrast [9]. Further, given that higher order SC diffusion processing remains relatively novel [55], optimization of distortion and motion correction should be considered. We also recognize that we did not use gadolinium contrast for the purpose of this investigation, which would allow for further characterization of lesion age or activity to more effectively link diffusion measures with tissue pathology or status.

## 5 Conclusion

In a novel study applying comprehensive brain and SC imaging in the RRMS population, we determined simple (i.e., DTI) and advanced (i.e., SMIfw) diffusion models are feasible for implementation, providing notable contrast between lesions and NAWM in pwRRMS and high reliability in HCs. Reduced axonal density and cellular integrity were observed as core features of MS pathology in MS lesions, though with divergent microstructural signatures in the brain and the cord. These alterations were likewise present in MS NAWM, suggestive of alterations in the tissue environment even before radiographic evidence of macrostructural changes. Both DTI and SMIfw were reliable across multiple scans for both the brain and SC. DTI measures in the brain were sensitive to detecting microstructural damage, while SMIfw measures like *f* performed better in the SC, supporting the notion that diffusion models may be better suited depending on the anatomic region. This work establishes a basis from which ongoing research may continue, with the goal of combining diffusion information with clinical measures to more effectively close the gap between advanced MRI imaging as a diagnostic tool and the presentation of clinical symptoms in pwMS.

## Author Contributions

K.G.S., S.B., and S.S. contributed to study conception, design, and acquisition of funding. A.A.W. contributed to data analysis and writing. I.S., G.S., A.E.C., A.A.W., and K.P.O. contributed to data acquisition. All authors contributed to editing and approving the final manuscript.

## Ethics Statement

All procedures performed in studies involving human participants were in accordance with the ethical standards of the institutional and/or national research committee. Informed consent was obtained from all individual participants included in the study.

## Conflicts of Interest

The authors declare no conflicts of interest.

## Supporting information


**FIGURE S1:** *β* effect size comparison of measures between HC WM and MS NAWM in the brain and SC. Measures and morphometry were *z*‐scored prior to analysis, with age, sex, and morphometry included as covariates in the linear model. Measures were grouped into broad categories based on what tissue characteristics the measure describes, including volume fraction, variance, or diffusivity. Significance was indicated by * (*p* < 0.050) following FDR correction. Diff., diffusion; EA, extra‐axonal; IA, intra‐axonal; *f*, neurite density fraction; *f*_*w*_, free‐water fraction.

**FIGURE S2:** *β* effect size comparison of measures between MS NAWM and MS lesions in the brain and SC. Measures and morphometry were *z*‐scored prior to analysis, with age, sex, and morphometry included as covariates in the linear model. Measures were grouped into broad categories based on what tissue characteristics the measure describes, including volume fraction, variance, or diffusivity. Significance was indicated by * (*p* < 0.050) following FDR correction.

**FIGURE S3:** Sensitivity‐reliability analysis in the brain using absolute *β* effect size between MS NAWM versus MS lesions and HC WM scan‐rescan ICC, with DTI, DKI, and SMIfw included as diffusion models. Significance is based on the FDR‐corrected *p*‐values from the absolute *β* effect size measurements.

**FIGURE S4:** Sensitivity‐reliability analysis in the SC using absolute *β* effect size between MS NAWM versus MS lesions and HC WM scan‐rescan ICC, with DTI, DKI, and SMIfw included as diffusion models. Significance is based on the FDR‐corrected *p*‐values from the absolute *β* effect size measurements.

## Data Availability

To abide by local institutional policies, a signed data transfer agreement will be required for access.
