# Supplementary figures and images for: Standard Model Imaging in the Brain and Spinal Cord of MS Patients: Initial Assessment and Comparison to Diffusion Tensor Imaging
Source: NMR Biomed. 2026 Jul 8;39(8):e70354. doi: 10.1002/nbm.70354 (PMC13346334; doi:10.1002/nbm.70354)

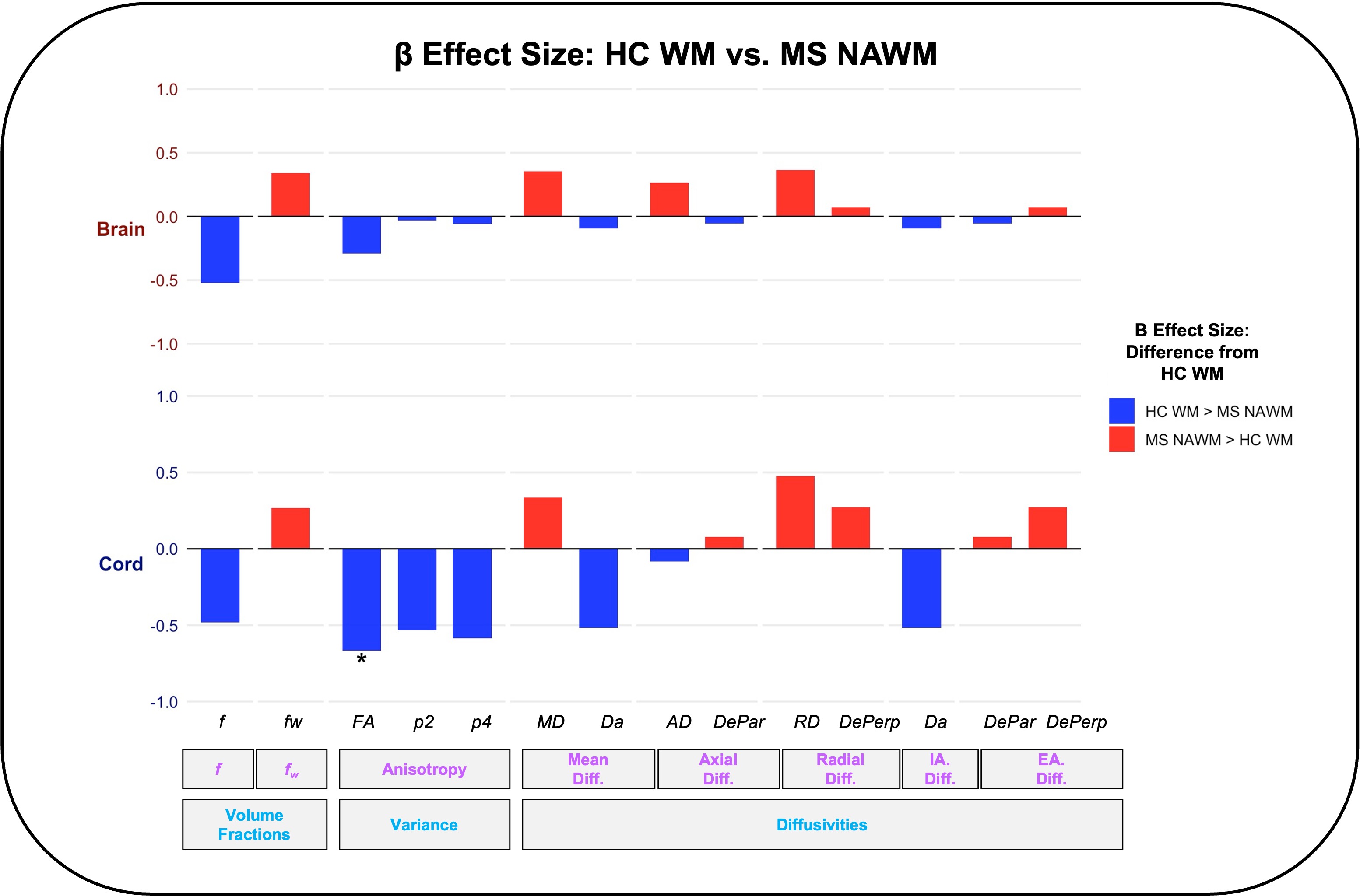

Supplement: Supplementary file 1 — FIGURE S1: β effect size comparison of measures between HC WM and MS NAWM in the brain and SC. Measures and morphometry were z‐scored prior to analysis, with age, sex, and morphometry included as covariates in the linear model. Measures were grouped into broad categories based on what tissue characteristics the measure describes, including volume fraction, variance, or diffusivity. Significance was indicated by * (p < 0.050) following FDR correction. Diff., diffusion; EA, extra‐axonal; IA, intra‐axonal; f, neurite density fraction; f w , free‐water fraction. [file NBM-39-e70354-s001.jpg]

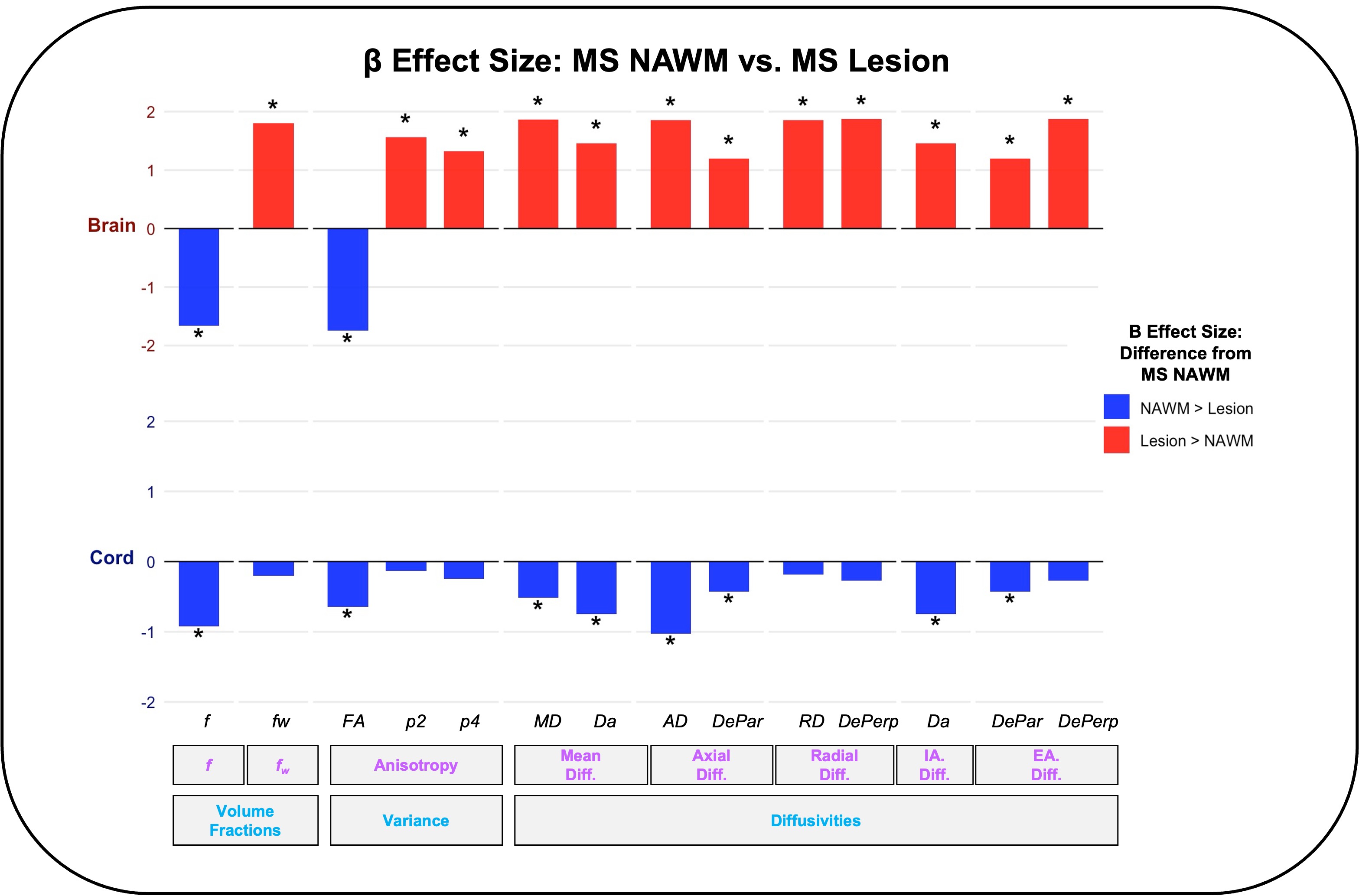

Supplement: Supplementary file 2 — FIGURE S2: β effect size comparison of measures between MS NAWM and MS lesions in the brain and SC. Measures and morphometry were z‐scored prior to analysis, with age, sex, and morphometry included as covariates in the linear model. Measures were grouped into broad categories based on what tissue characteristics the measure describes, including volume fraction, variance, or diffusivity. Significance was indicated by * (p < 0.050) following FDR correction. [file NBM-39-e70354-s004.jpg]

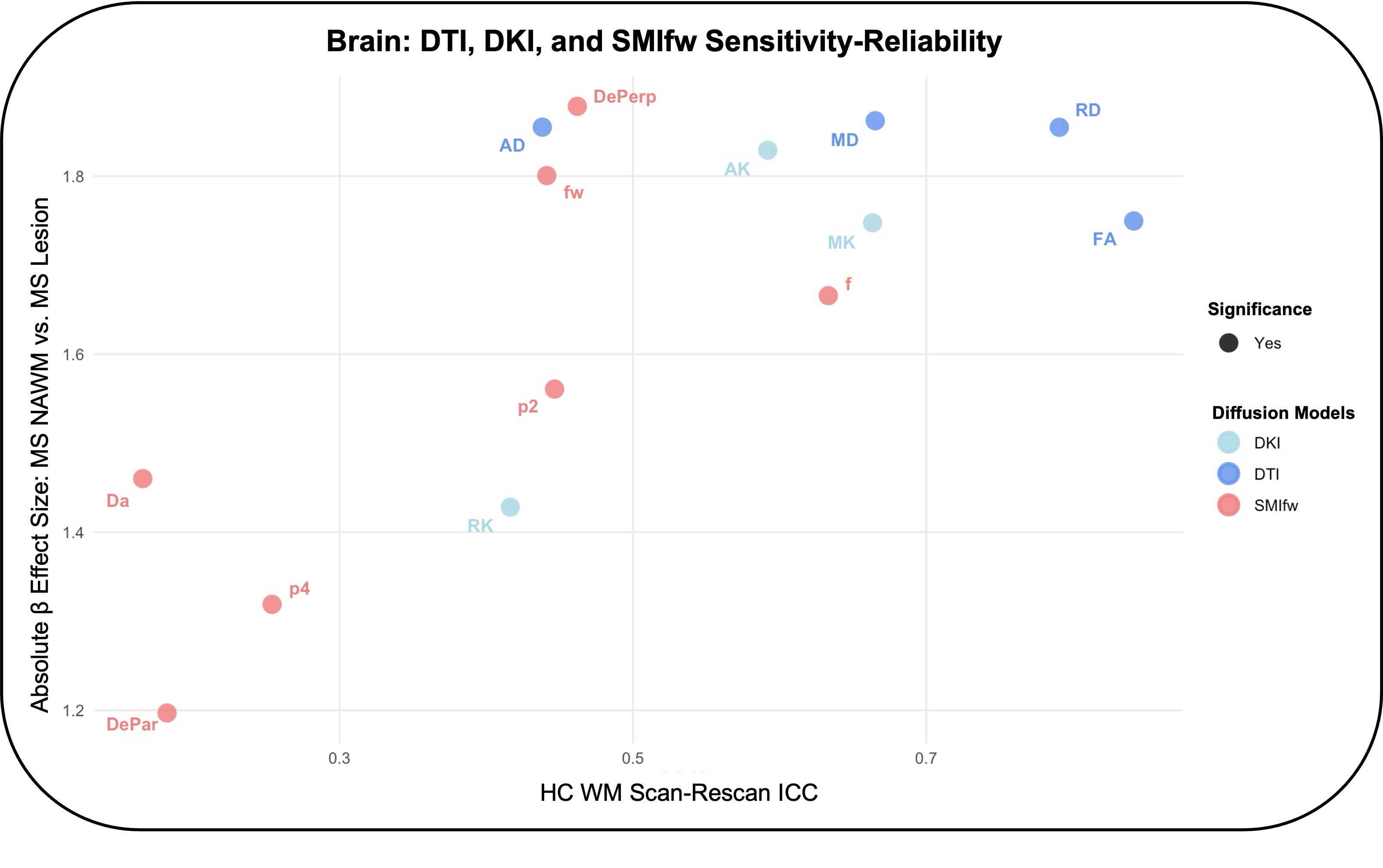

Supplement: Supplementary file 3 — FIGURE S3: Sensitivity‐reliability analysis in the brain using absolute β effect size between MS NAWM versus MS lesions and HC WM scan‐rescan ICC, with DTI, DKI, and SMIfw included as diffusion models. Significance is based on the FDR‐corrected p‐values from the absolute β effect size measurements. [file NBM-39-e70354-s002.jpg]

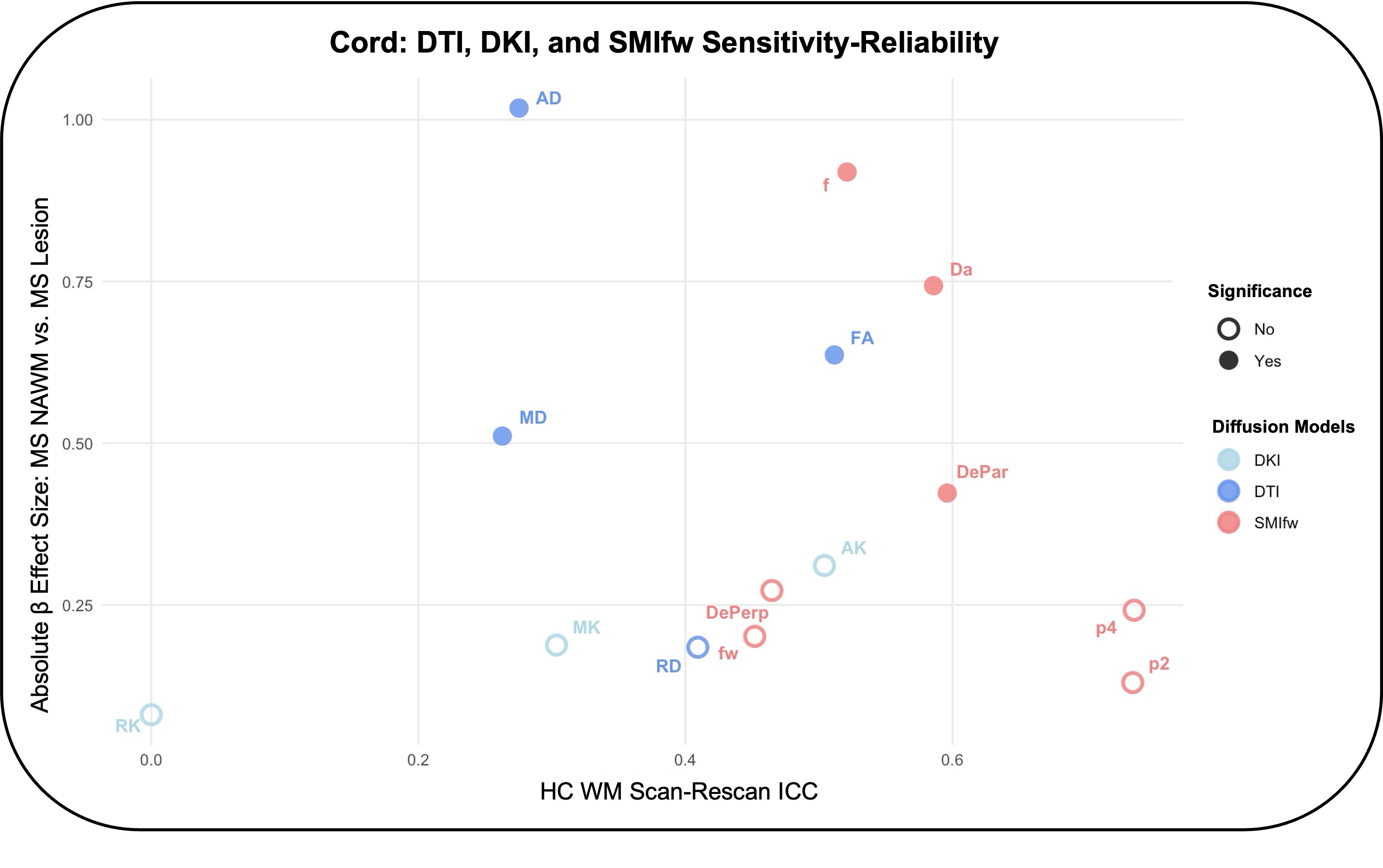

Supplement: Supplementary file 4 — FIGURE S4: Sensitivity‐reliability analysis in the SC using absolute β effect size between MS NAWM versus MS lesions and HC WM scan‐rescan ICC, with DTI, DKI, and SMIfw included as diffusion models. Significance is based on the FDR‐corrected p‐values from the absolute β effect size measurements. [file NBM-39-e70354-s003.jpg]
